# Older adults make sense of their suicidal behavior: a Swedish interview study

**DOI:** 10.1093/geroni/igaf122.2709

**Published:** 2025-12-31

**Authors:** Sara Hed, Anne Ingeborg Berg, Stefan Wiktorsson, Jennifer Strand, Silvia Sara Canetto, Margda Waern

**Affiliations:** University of Gothenburg, Gothenburg, Vastra Gotaland, Sweden; University of Gothenburg, Gothenburg, Vastra Gotaland, Sweden; University of Gothenburg, Gothenburg, Vastra Gotaland, Sweden; University of Gothenburg, Gothenburg, Vastra Gotaland, Sweden; Colorado State University, Fort Collins, Colorado, United States; University of Gothenburg, Gothenburg, Vastra Gotaland, Sweden

## Abstract

This study explored how older adults (70+) understood a recent suicidal act, and what changed in the aftermath. Four women and five men (71-91 years) receiving care at a geriatric psychiatric outpatient clinic in Sweden took part in two interviews. Most of the women and none of the men had engaged in prior suicidal acts. Interpretative phenomenological analysis was employed. The suicidal act was explained as a response to losses in physical and cognitive functions, social roles and relationships that rendered previous coping strategies unviable. Participants reported being dependent on a healthcare system that they experienced as indifferent and even dismissive of their suffering. The suicidal act was described as an unplanned act of despair. Positive changes followed for participants who reported having had suicidal ideation prior to the suicidal act and had insights into its triggers. Some gained access to needed medical care; others developed greater awareness of their psychological needs and improved coping skills. Individuals who said that they had not had suicidal thoughts prior to the suicidal act and could not explain it reported no positive change in the aftermath. Participants’ age-related losses were in many cases exacerbated by negative interactions with health care providers, indicating that continued attention needs to be given to implicit ageism in medical professionals. The suicidal acts were described as impulsive, which was unexpected because a dominant belief is that older adult suicidal behavior is planned. Older adult suicide planning should be addressed in larger studies across geographical and cultural settings.

